# Is satisfaction a direct predictor of nursing turnover? Modelling the relationship between satisfaction, expressed intention and behaviour in a longitudinal cohort study

**DOI:** 10.1186/1478-4491-6-22

**Published:** 2008-10-31

**Authors:** Trevor Murrells, Sarah Robinson, Peter Griffiths

**Affiliations:** 1 National Nursing Research Unit, King's College London, Florence Nightingale School of Nursing and Midwifery, 57 Waterloo Road, London, SE1 8WA, UK

## Abstract

**Background:**

The theory of planned behaviour states that attitudinal variables (e.g. job satisfaction) only have an indirect effect on retention whereas intentions have a direct effect. This study uses secondary data from a longitudinal cohort of newly qualified nurses to test for the direct and indirect effects of job satisfaction (client care, staffing, development, relationships, education, work-life interface, resources, pay) and intentions to nurse on working as a nurse during the 3 years after qualification.

**Methods:**

A national sample (England) of newly qualified (1997/98) nurses (n = 3669) were surveyed at 6 months, 18 months and 3 years. ANOVA and MANOVA were used for comparison of mean job satisfaction scores between groups; intentions to nurse (very likely, likely vs. unlikely, very unlikely and unable to say at this stage); working (or not working as a nurse) at each time-point. Indirect and direct effects were tested using structural equation and logistic regression models.

**Results:**

Intentions expressed at 6 months to nurse at 18 months were associated with higher scores on pay and relationships, and intentions at 3 years were associated with higher scores on care, development, relationships, work-life interface, resources, pay respectively. Intentions expressed at 18 months to nurse at 3 years were associated with higher scores on development, relationships, education and work-life interface. Associations with actual nursing were fewer. Those working as a nurse had higher satisfaction scores for development (18 months) and relationships (3 years). Regression models found significant associations between the pay and staffing factors and intentions expressed at 6 months to nurse at 18 months, and between pay and intentions to nurse at 3 years. Many of the associations between intentions and working as a nurse were significant. Development was the only job satisfaction factor significantly associated with working as a nurse and just at 18 months.

**Conclusion:**

Results partially support the theory of planned behaviour. Intentions expressed by nurses are stronger predictors of working as a nurse than job satisfaction. Retention strategies should focus on identifying nurses showing early signs of departure with emphasis on developmental aspects, mentoring and support.

## Background

Job satisfaction is a major feature of nursing turnover research [1] more so than perhaps any other factor and is frequently used to predict turnover. However, the theory of planned behaviour [2] postulates that attitudes towards behaviour, subjective norms and perceptions of behavioural control have a direct effect on intentions but an indirect effect on actual behaviour, mediated through intentions, on actual behaviour (i.e. attitudes affect intentions which then impact on behaviour). Since job satisfaction scales largely comprise attitudinal items the same relationship would be expected to apply to the link between satisfaction and turnover and thus satisfaction is only an indirect predictor.

The theory of planned behaviour evolved from the consistent finding that attitudes were poor predictors of behaviour in many circumstances [3] and proposes that people act in accordance with their intentions and perceptions of control over behaviour. Behaviours can be predicted from intentions with considerable accuracy [4] when control is not overly constrained. Intentions in turn are influenced by attitudes toward the behaviour, subjective norms, and perceptions of behavioural control. The theory identifies three independent determinants of intention: attitude towards behaviour, subjective norm and lastly perceived behavioural control. The first determinant reflects how much an individual has a favourable evaluation of the behaviour, the second is a reflection of the social pressure to perform the behaviour and the third represents the perceived ease or difficulty of performing the behaviour. The theory begins with the determinants of these antecedents and proposes that behaviour is a function of salient information, or beliefs, relevant to the behaviour. Three salient beliefs are identified: *behavioural beliefs *that influence attitudes towards behaviour, *normative beliefs *that constitute the underlying determinants of subjective norms, and *control beliefs *that provide the basis for perceptions of behavioural control.

Two major reviews of nurse turnover [5] and job satisfaction [1] have been conducted recently. An earlier meta-analysis on job satisfaction and turnover of nurses [6] found a strong positive relationship between behavioural intentions and turnover, a strong negative relationship between job satisfaction and behavioural intentions and a small negative relationship between job satisfaction and turnover that provides support for the theory of planned behaviour. The Lu *et al*. [1] review found that job satisfaction, whether used as a single generic measure or as a number of component measures, was a significant predictor of turnover and intention-to-quit. Specific components of job satisfaction that were associated with retention factors included work overload, rotating shifts, interpersonal relationships/group cohesion, kinship responsibility, promotion/opportunities, pay, autonomy and job stress. Findings, however, were not always consistent.

Turnover behaviour often emerges as a multistage process that includes attitudinal, decisional and behavioural components [7]. Job satisfaction was found to have only an indirect effect on turnover in a model exploring the causal pathways between pay satisfaction, job satisfaction, organizational commitment and turnover intent [7]. The overall conclusion was that nurses, who are satisfied with job and pay, are committed to the organization, and less likely to leave voluntarily.

Job satisfaction has been linked to thinking about quitting and intention to search for another job but not to actual turnover of hospital employees [8]. Others dispute the idea that intentions are the best predictors of turnover and believe that intentions have been confused with expectations [9,10]. The closeness in time between intentions expressed and turnover has been found to contribute to the successful identification of associations [11].

This study uses secondary data from a nationally representative (England) longitudinal cohort of nurses who qualified from the diploma programme in 1997/1998 to test the hypotheses that job satisfaction has an indirect effect, mediated through intentions, and has a direct effect on whether a recently qualified nurse was nursing at 18 months and three years after qualification. The direct effects of intentions on actual nursing are also tested.

## Methods

### Research design

The research design was correlational and longitudinal. Subjects were surveyed prospectively from qualification onwards and at three subsequent time-points (6 months, 18 months and 3 years).

### Research hypotheses

#### Primary hypotheses

1. Self-reported job satisfaction predicts intentions expressed about working as a UK nurse.

2. Self-reported job satisfaction at earlier time-points (6 months, 18 months) predicts working as a UK nurse at 18 months and 3 years after qualification.

3. Intentions expressed at earlier time-points predict working as a UK nurse at 18 months and 3 years.

If only 1 and 3 are satisfied then self-reported job satisfaction only has an indirect effect on working as a UK nurse and therefore supports the theory of planned behaviour. If 1, 2 and 3 are satisfied then self-reported job satisfaction has both a direct and indirect (mediated through intentions) effect on working as a nurse and only partially supports the theory.

#### Secondary hypothesis

Prior intentions predict intentions expressed at subsequent time-points.

### Sample selection

The study population consisted of all qualifiers from the adult, child and mental health branches of the diploma programme in England in 1997/98. A full census was taken for the child branch because of its smaller size (N = 986 based on English National Board figures [12]) and because pilot work had shown that recruitment rates were lower for this branch. The estimated size of adult (N = 7214) and mental health branches were larger (N = 1396) therefore nurses were sampled from both populations. Strata were formed from eight regional health authorities (RHA) each containing a variable number of colleges. From each region a half of adult branch and two-thirds of mental health branch colleges, or the nearest fraction above a half/two-thirds, were selected from each region. There was further sub-sampling of intakes from larger colleges of the adult branch. The total number eligible to participate was 3669 and 3213 nurses were recruited [12]. Response rates to the at qualification, 6 month, 18 month and 3 year questionnaires were 76% (2784), 64% (2331), 53%(1957) and 45% (1651).

A postal questionnaire was used for data collection. A number of strategies were adopted to maintain response rates. Nurses who attended face-to-face recruitment sessions prior to qualification provided contact addresses (home address and an alternative, typically parents address) which allowed regular contact. Questionnaires were sent twice to the main address if no response to the first mail-out, and on a third occasion to the alternative address. If no response after the three mailings nurses were traced via the United Kingdom Central Council (UKCC) for Nursing, Midwifery and Health Visiting (now the Nursing and Midwifery Council) and a questionnaire was sent on our behalf by the UKCC.

### Job satisfaction instrument

A job satisfaction question was developed for the study, as part of a larger questionnaire, and psychometrically tested on the adult, child, learning disability and mental health branches[13]. The learning disability branch did not produce a consistent structure across time or with other branches and for this reason they were excluded from further psychometric testing. Seven components (factors), were identified: client care, staffing, development, relationships, education, work-life interface, resources). The items that loaded under each factor are shown in Table 1.

**Table 1:** Measurement model

**Factor**	**Item**
**Client Care**	Proportion of time I spend/spent providing direct client care ('hands on' care)
	Opportunities to provide good quality care
	Proportion of time I spend/spent on paperwork
	
**Staffing**	Ratio of qualified to unqualified staff on days
	Number of staff usually on days
	
**Development**	Opportunity to reflect on my practice with someone of a higher grade/position
	Opportunity to reflect on practice with a group of colleagues
	Opportunity to reflect on my own practice on my own while at work
	Frequency of discussions about developing my career
	Constructive feedback on my work from staff of a higher grade/position
	Emotional support from my immediate line manager
	
**Relationships**	Quality of working relationships with colleagues
	Emotional support from nurses of the same grade/position
	
**Education**	Opportunitiy to go on courses other than study days/workshops
	Opportunity to go on study days/workshops
	
**Work-Life Interface**	Notice of off duty
	Combining work hours with social life
	Frequency with which I leave work on time
	
**Resources**	
*Adult and Child*	Availability of equipment(e.g. hoists)
	Availability of supplies (e.g. dressings)
	
*Mental Health*	Availability of equipment (e.g. audiovisual, art materials, books)
	Availability of facilities (e.g. day room, quiet room, interview room)
	
**Pay**	Pay in relation to level of responsibility

The items that loaded under each factor were consistent across the three remaining branches and time (6 months, 18 months, 3 years) and factors had good internal reliability (Cronbach's α Adult 0.62–0.92; Child 0.59–0.89; Mental health 0.57–0.92). Internal reliability was lowest for work-life interface (0.57–0.66) and highest for education (0.88–0.92). The job satisfaction question also contained two items related to pay and grade. The latter was not asked at 6 months because most nurses were still in D grade posts. Consequently the remaining item on pay was excluded from the psychometric testing because it would result in a single factor however because pay is so important it has been included in the modeling.

Until December 2004 newly qualified nurses in the UK were typically appointed to a D Grade post and after a minimum of 6 months post-registration experience would be able to apply for an E grade post. At this grade nurses were often encouraged to gain valuable management experience and/or receive further training in a specialty (e.g. accident and emergency). An increased managerial role was expected of those appointed to F grade posts and these nurses were sometimes left in charge of a ward or other setting. In December 2004 these grades were superseded in the UK by Agenda for Change pay bands [14].

### Key variables

#### Intentions to work as a nurse in the future

Respondents were asked for example "how likely is it that you will be working in nursing or health care in the UK 18 months from now?" and could respond very likely, quite likely, unlikely, very unlikely or unable to say at this stage. A decision was taken to reduce this categorization into two groups: likely (very likely, quite likely) and unlikely/uncertain (unlikely, very unlikely or unable to say).

#### Working as a nurse

A career chart was used to determine whether or not a respondent was working in a nursing post or as an agency or bank nurse at a particular time-point. On the chart the respondent would provide information on all nursing jobs, other health care jobs, agency/bank work, maternity leave, full-time courses, unemployment, working abroad etc [15]. Each line on the chart would have an event number and a start and end date. Additional information was requested for nursing jobs, which included location, employing organization, specialty, grade and type of contract (established or temporary post). Events at 6 months, 18 month and 3 years were extracted from the career chart and the activity code for the event was used to classify events into nursing jobs and all other activities.

### Data analysis

Basic statistics (percentages, means) were computed to show whether relationships existed between job satisfaction, intentions and working in nursing at 18 months and 3 years. Tetrachoric rather than Pearson correlation coefficients were used to measure association between binary variables (intentions, nursing). A factor analysis was conducted in SPSS version 15 on job satisfaction data at 6 and 18 months using principal component analysis with varimax rotation and Kaiser normalization to ascertain whether the eight factors (Care, Staffing, Development, Relationships, Education, Work-Life Interface, Resources and Pay) loaded on one or more second-order or higher level factors. In both cases two factors were identified.

Analysis of Variance (ANOVA) and Multivariate Analysis of Variance (MANOVA) were used to test for statistical difference in first-order job satisfaction factors between two independent groups (see Key variables above). Single dependent variables were analyzed using ANOVA and multiple dependent variables that loaded under the same factor were analyzed using MANOVA. These two tests provide useful preliminary information before fitting the statistical models described below. High correlation was found between intentions variables that were asked at the same time-point. The tetrachoric correlations between intentions expressed at qualification (looking ahead to the 6 months, 18 month and 3 year time-points) ranged from 0.820 (95% CI 0.767 to 0.873) to 0.964 (95% CI 0.946 to 0.982), and the correlation between intentions expressed at 6 months looking ahead to 18 months and 3 years was 0.878 (95% CI 0.841 to 0.915). Due to high correlations, and to avoid collinearity, a decision was taken to include just those intentions variables where the time-point in the future matched with the dependent variable. For example in the model of nursing at 3 years we included intentions at qualification looking ahead to 3 years but not to 6 and 18 months. Similarly we included intentions at 6 months looking ahead to 3 years but not to 18 months. Tetrachoric correlations between successive pairs of working in nursing variables were not as strong and ranged from 0.609 (95% CI 0.519 to 0.699) between qualification and 6 months to 0.073 (95% CI 0 to 0.200) between qualification and 3 years. These variables were therefore retained in all models where they were antecedent to the dependent variable of interest. Therefore in the model for nursing at 3 years we include variables for working in nursing at qualification, 6 months and 18 months.

The research hypotheses were tested using structural equation modeling (SEM) and logistic regression. SEM estimates regression coefficients between latent (unobserved) and observed variables that minimize the difference between the covariance structure of the observed data and the predicted model. Logistic regression models predict the occurrence of a binary event using a number of independent variables. SEM was used when job satisfaction was represented by two second order constructs and logistic regression when individual first-order job satisfaction factors were used as independent variables in the model. Each model included seven moderator variables: branch (Adult, Child, Mental Health), age, gender, partner, children (living at home), ethnicity (four groups: white British, white Irish, other white nationalities, Black, Asian and Chinese) and highest educational qualification (five groups: Degree, Sufficient for degree entry (2 A-levels or more), not sufficient for degree entry, Access course or DC test, Other).

The literature on SEMs is extensive [16-18] but less so for SEMs involving binary dependent variables [19-24]. We used Mplus developed by Muthén and Muthén [25] for both SEM and logistic regression modelling. The program has the ability to model missing data in the dependent variable under certain conditions. The Mplus program produces standard output that includes parameter estimates (β), standard errors (se(β)), Z test (β/se(β)) for parameters (paths, intercepts, correlations, variances and residual variances), the log likelihood and a limited number of measures of fit (Akaike Information Criterion, Bayesian Information Criterion). The global effect of first order factors that loaded under each second-order factor were tested using the Wald χ^2 ^statistic in the logistic regression model.

There were an insufficient numbers of respondents for a robust analysis of each branch separately using the methods described above so respondents from all three branches were amalgamated into one dataset. Branch was included as an independent variable in the statistical models and was a significant predictor in just one model where working as a nurse at 18 months was the dependent variable. In that model adult and child branch nurses were less likely to be nursing at 18 months than mental health nurses.

Job satisfaction trends were found to vary across branch and time for this sample [26]. The level of job satisfaction and the ranking of components were on the whole similar for the adult and child branches but different for mental health.

### Ethical considerations

This study pre-dated the requirement of MREC approval, guidance was followed from staff of the university from which students were recruited as to the internal procedures required for ethical approval. At no time were participants or Colleges identified. The only addresses held on local databases were those provided by the participant.

## Results

The data support the secondary hypothesis that prior intentions predict intentions expressed at subsequent time-points. Most nurses (95%, 1998) who at qualification said they were intending nurse in the UK at 18 months expressed the same intention at 6 months whereas 66% (73) changed their intentions from unlikely or uncertain (at qualification) to likely (at 6 months). The corresponding figures looking a head to 3 years were 91% (1783) and 65% (167) respectively. Intentions expressed at qualification and 18 months (92%, 1543 vs. 66%, 143), and 6 months and 18 months (92%, 1555 vs. 60%, 134) about working in UK nursing at 3 years produced similar findings. All these associations were statistically significant (Fisher's Exact Test p < .001).

Before testing hypotheses involving job satisfaction a decision was taken to second order factor analyse the instrument scores since we could not assume that these factors were the manifestation of a single underlying latent variable. Factor analysis of the 6-month data identified two second-order factors. Client care (rotated loading 0.73), Staffing (0.68), Work-Life Interface (0.56), Resources (0.65) and Pay (0.73) loaded on second-order factor 1(SF1) (variance explained (VE) 30%) and Development (0.79), Relationships (0.81) and Education (0.73) on second-order factor 2 (SF2) (VE 28%). Analysis of the 18-month data identified the same two factors, SF1 (VE 31%): Client care (0.75), Staffing (0.74), Work-Life Interface (0.59), Resources (0.60) and Pay (0.71) and SF2 (VE 26%): Development (0.78), Relationships (0.76) and Education (0.76). In both cases (6 months, 18 months) the Kaiser-Meyer-Olkin measure of sampling adequacy was good (0.87, 0.85), Bartlett's Test of Sphericity was statistically significant (p < .001) and loadings were similar.

A two-group comparison (unlikely/uncertain vs. likely) of mean job satisfaction scores for intentions to nurse at 18 months as expressed at 6 months was used initially to test the primary hypothesis 1 that job satisfaction and intentions to work as a nurse were associated. Mean scores differed significantly on two first-order factors: Pay (2.28 vs. 2.55, p = .022) and Relationships (3.88 vs. 4.03, p = . 040) and differences approached statistical significance (p < .10) on another three: Development (2.88 vs. 3.05, p = .057), Work-Life Interface (3.29 vs. 3.45, p = .065) and Resources (3.35 vs. 3.52, p = .078) and were similar for the remaining three factors: Client care (3.16 vs. 3.25, p = .31), Staffing (3.33 vs. 3.28, p = .61) and Education (3.22 vs. 3.16, p = .63). First-order factors loading under each second-order factor also differed significantly between intention groups in the MANOVA (SF1: F_5,2116 _= 2.361, p = .038 SF2: F_3,2143 _= 2.682, p = .045).

Differences were more striking for intentions to nurse at 3 years. At 6 months mean scores differed significantly on six factors: Care (3.09 vs. 3.27, p = .007), Development (2.90 vs. 3.06, p = .012), Relationship (3.90 vs. 4.03, p = 0.13), Work-Life Interface (3.26 vs. 3.47, p = .002), Resources (3.31 vs. 3.54, p = .002) and Pay (2.28 vs. 2.57, p = .001) and were similar for the other two: Staffing (3.18 vs. 3.30, p = .13) and Education (3.08 vs. 3.18, p = .25). First-order factors loading under each second-order factors differed significantly between intention groups (SF1: F_5,2113 _= 2.510, p < .001 SF2: F_3,2140 _= 2.619, p = .049). At 18 months mean scores differed significantly on four factors: Development (2.90 vs. 3.14, p = .003), Relationships (3.81 vs. 4.01, p = .003), Education (3.19 vs. 3.54, p = .001) and Work-Life Interface (3.25 vs. 3.51, p = .001), approached significance on another one: pay (2.52 vs. 2.72, p = .068) and were similar for the remaining three: Client care (3.25 vs. 3.37, p = .14), Staffing (3.19 vs. 3.31, p = .22) and Resources (3.47 vs. 3.59, p = .19) and differed significantly in the two MANOVAs (SF1: F_5,1631 _= 2.510, p = .028 SF2: F_3,1714 _= 4.965, p = .002).

A comparison of mean job satisfaction scores between those who were not working as a nurse and those who were working as a nurse at 18 months was used initially to test the primary hypothesis 2 that job satisfaction was associated with working as a nurse. Mean scores differed significantly on one first-order factor only: Development (2.84 vs. 3.05, p = 0.008) and mean scores were similar for all other first order factors: Client care (3.27 vs. 3.25, p = .81), Staffing (3.21 vs. 3.28, p = .42), Relationships (3.98 vs. 4.02, p = .54), Education (3.09 vs. 3.18, p = .43), Work-Life Interface (3.37 vs. 3.44, p = .33), Resources (3.52 vs. 3.51, p = .89), Pay (2.48 vs. 2.55, p = .51). Only those factors loading onto SF2 differed significantly between intention groups (SF1: F_5,1831 _= 0.566, p = .73 SF2: F_3,1850 _= 2.888, p = .034).

Only one difference emerged for working as a nurse at 3 years for Relationships (3.87 vs. 4.01, p = .041). All other first order factors did not differ significantly: Client care (3.37 vs. 3.35, p = .74), Staffing (3.33 vs. 3.27, p = .56), Development (3.05 vs. 3.13, p = .32), Education (3.40 vs. 3.52, p = .24), Work-Life Interface (3.44 vs. 3.48, p = .60), Resources (3.48 vs. 3.56, p = .39) and Pay (2.60 vs. 2.75, p = .18). Intention groups did not differ significantly in either of the MANOVAs (SF1: F_5,1316 _= 0.667, p = .67 SF2: F_3,1385 _= 1.190, p = .31).

All three intentions to work as a nurse variables were associated with working as a nurse at 18 months (Fisher's Exact test p < .001) and all six intentions variables were associated with working as a nurse at 3 years (Fisher's Exact Test p < .001)(Table 2) and therefore primary hypothesis 3 was supported.

**Table 2:** Intentions and nursing at 18 months and 3 years

		**18 months**				**3 years**			
		**Not nursing**		**Nursing**		**Not nursing**		**Nursing**	
	**Likelihood of nursing**	No.	%	No.	%	No.	%	No.	%
**At qualification looking ahead to:**									
6 months	Unlikely/Uncertain	17	26	48	74	16	35	30	65
	Likely	161	9	1679	91	193	13	1300	87
18 months	Unlikely/Uncertain	28	31	63	69	25	37	42	63
	Likely	150	8	1667	92	184	12	1289	88
3 years	Unlikely/Uncertain					45	27	122	73
	Likely					165	12	1209	88
									
**At 6 months looking ahead to:**									
18 months	Unlikely/Uncertain	38	33	77	67	28	33	56	67
	Likely	137	8	1663	92	182	12	1281	88
3 years	Unlikely/Uncertain					44	27	119	73
	Likely					165	12	1217	88
									
**At 18 months looking ahead to:**									
3 years	Unlikely/Uncertain					64	39	100	61
	Likely					148	11	1260	89

Intentions accurately predict working as a nurse in the future for those who state very likely or likely (87–92%) but was less effective at predicting those not working as a nurse in the future amongst those who stated very unlikely, unlikely or unable to say at this stage (26 – 39%).

### Statistical modelling

The final stage of analysis focuses on the modelling of intentions and working as a nurse. The relationships between intentions expressed at earlier time-points and current intentions were all statistically significant (Table 3) and therefore the secondary hypothesis was supported.

**Table 3:** Intentions to work as a nurse in the future : SEM and logistic regression models

	**Model 1**	**Model 2**	**Model 3**
**Surveyed at:**	6 months	6 months	18 months
**Likelihood of nursing at:**	18 months	3 years	3 years
	OR (95% CI)	OR (95% CI)	OR (95% CI)
*Structural Equation Model*	(n = 2238)	(n = 2239)	(n = 1860)
**6 months:**			
JS – Factor 1	1.11 (0.59 – 2.12)	1.58 (0.99 – 2.53)	
JS – Factor 2	1.20 (0.70 – 2.05)	0.96 (0.65 – 1.44)	
			
**18 months:**			
JS – Factor 1			0.89 (0.52 – 1.54)
JS – Factor 2			1.54 (0.97 – 2.44)
			
**At qualification:**			
LN at 18 months	7.85 (4.90 – 12.60)^c^		
LN at 3 years		4.44 (3.22 – 6.11)^c^	2.99 (2.02 – 4.41)^c^
			
**At 6 months:**			
LN at 3 years			5.11 (3.53 – 7.41)^c^
			
**Nursing at:**			
6 months	3.88 (2.20 – 6.87)^c^	2.88 (1.72 – 4.81)^c^	
18 months			4.71 (3.12 – 7.10)^c^
			
*Logistic regression*	(n = 2045)	(n = 2045)	(n = 1553)
			
**JS 6 months/18 months**			
			
*Factor 1 (X*^2^, *p) 5df*	(10.624, .059)	(15.102, .010)^a^	(2.650, .75)
Care	0.94 (0.72 – 1.23)	1.00 (0.82 – 1.21)	0.86 (0.65 – 1.14)
Staffing	0.79 (0.62 – 1.00)^a^	0.92 (0.78 – 1.09)	1.06 (0.84 – 1.33)
W-L Balance	1.20 (0.92 – 1.55)	1.18 (0.97 – 1.42)	1.15 (0.89 – 1.49)
Resources	1.31 (0.91 – 1.40)	1.13 (0.97 – 1.32)	0.96 (0.77 – 1.19)
Pay	1.21 (1.01 – 1.45)^a^	1.18 (1.03 – 1.35)^a^	1.01 (0.84 – 1.21)
*Factor 2 (X*^2^, *p) 3df*	(4.343, .23)	(0.671, .88)	(7.010, .072)
Development	1.16 (0.87 – 1.55)	1.00 (0.81 – 1.24)	1.22 (0.92 – 1.64)
Relationships	1.18 (0.87 – 1.60)	1.09 (0.87 – 1.37)	1.00 (0.74 – 1.37)
Education	0.86 (0.72 – 1.04)	1.00 (0.87 – 1.14)	1.15 (0.96 – 1.39)

a < .05; b < .01; c < .001

JS = Job satisfaction; LN = Likelihood of nursing

Whether a respondent was working as a nurse or not was also significantly associated with intentions expressed at earlier time-points and therefore supports primary hypothesis 3.

The evidence supporting an association between job satisfaction and intentions is conflicting (primary hypothesis 1). At 6 months looking ahead to 18 months neither of the second-order factors was associated with intentions in the SEM whereas the logistic regression found significant associations for Staffing and Pay. The global effect of Care, Staffing, Work-Life Interface, Resources and Pay however falls short of statistical significance (p = .059). An odds ratio (OR) less than one for the Staffing was an unexpected finding. Tolerance, defined as the amount of variation not explained by the other seven job satisfaction factors, ranged from 0.57 to 0.79 amongst the job satisfaction factors therefore the finding for Staffing cannot be attributed just to collinearity and, as was shown previously, satisfaction with staffing was marginally lower for those who were likely to nurse at 18 months than those who were unlikely or unable to say at this stage when asked at qualification (3.28 vs. 3.33).

Greater consistency emerges between the SEM and logistic regression models when nurses were asked to look ahead to the 3-year time-point. The association between SF1 and intentions at 3 years for the 6-month data was significant (OR 1.56 95% CI 1.02 – 2.48) in the model that did not include prior intentions and working as a nurse at 6 months and was close to statistical significance in the full model (Table 3). The global test of the five first-order factors loading on SF1 was statistically significant. The OR for Staffing was less than one again but was not significant. Satisfaction with Pay was significantly and positively associated with intentions to nurse. Positive associations emerged for Work-Life Interface and Resources without quite reaching conventional significance (Z = 1.67 and 1.55). There is some evidence for supporting primary hypothesis 1 but findings are not conclusive.

SF2 had a stronger association than SF1 with intentions at 3-years in the model for the 18-month time point. SF2 was significantly associated with intentions in the model that excluded prior intentions and working as a nurse at 18 months (OR 1.76 95%CI 1.12 – 2.76) and close to significance in the full model (Table 3). These findings were supported by the logistic regression global tests for first order factors loading on SF1 (p = .75) and SF2 (p = .072). The evidence on this occasion for supporting primary hypothesis 1 is less strong.

SEM is able to include more respondents in the model because each first-order job satisfaction factor is treated as a dependent variable and is modelled under the missing at random assumption (MAR) whereas in the logistic regression model these factors are treated as independent variables. The number of nurses can be increased in the logistic regression analysis by the simultaneous regression of the intentions and working as a nurse variables on their antecedents (where appropriate) and the baseline moderators. Using this approach it is possible to increase all the analysis samples from 2045 to 2238/2039 and 1553 to 1820. The findings for job satisfaction in these models remain largely unaltered.

Intentions to nurse at 18 months, as expressed at both qualification and 6 months were both positively associated with working as a nurse at 18 months (Table 4) and therefore primary hypothesis 3 is supported.

**Table 4:** Working as a nurse : SEM and logistic regression models

	**Model 1**	**Model 2**
**Nursing at:**	18 months	3 years
	OR (95% CI)	OR (95% CI)
*Structural Equation Model*	(n = 2136)	(n = 1780)
		
**6 months:**		
JS – Factor 1	0.70 (0.39 – 1.28)	
JS – Factor 2	1.45 (0.88 – 2.39)	
		
**18 months:**		
JS – Factor 1		0.67 (0.39 – 1.17)
JS – Factor 2		1.45 (0.91 – 2.30)
		
**At qualification:**		
LN at 6 months		
LN at 18 months	2.49 (1.38 – 4.57)^b^	
LN at 3 years		1.51 (0.94 – 2.41)
		
**At 6 months:**		
LN at 18 months	4.01 (2.39 – 6.71)^c^	
LN at 3 years		1.43 (0.88 – 2.33)
		
**At 18 months**		
LN at 3 years		3.19 (2.06 – 4.94)^c^
		
**Nursing at:**		
Qualification	1.17 (0.69 – 1.98)	0.71 (0.40 – 1.26)
6 months	7.62 (4.20 – 13.83)^c^	2.72 (1.35 – 5.50)^b^
18 months		4.18 (2.72 – 6.44)^c^
		
*Logistic regression*		
		
**JS 6 months/18 months**	(n = 1713)	(n = 1235)
		
*Factor 1 (X*^2^, *p) 5df*	(1.795, .88)	(3.869, .57)
Care	0.86 (0.67 – 1.11)	0.96 (0.73 – 1.27)
Staffing	1.03 (0.84 – 1.28)	0.84 (0.67 – 1.06)
W-L Balance	0.97 (0.76 – 1.24)	0.98 (0.76 – 1.27)
Resources	1.03 (0.85 – 1.25)	1.11 (0.90 – 1.38)
Pay	0.98 (0.83 – 1.16)	1.06 (0.89 – 1.27)
*Factor 2 (X*^2^, *p) 3df*	(9.498, .023)^a^	(0.647, .89)
Development	1.50 (1.15 – 1.97)^b^	1.09 (0.82 – 1.44)
Relationships	0.75 (0.56 – 1.01)	0.98 (0.72 – 1.33)
Education	0.98 (0.83 – 1.16)	1.03 (0.85 – 1.24)

a < .05; b < .01; c < .001

JS = Job satisfaction; LN = Likelihood of nursing

Second order factors were not associated with nursing at 18 months whereas the global test of first order factors loading on SF2 on nursing at 18 months was statistically significant (p = .023) and can be attributed to the positive association with Development and negative association with Relationships. These two first-order factors appear to have a counterbalancing effect on working as a nurse at 18 months. There is some evidence for supporting primary hypothesis 2 but it is not strong. All intentions except those expressed at 18 months looking ahead to 3 years were not significantly associated with working as a nurse at 3 years so the evidence is conflicting in regard to primary hypothesis 3 although one would expect this latter variable to have the strongest effect because of the shorter time lapse. Neither the second order nor the first order job satisfaction factors were associated with working as a nurse at 3 years and therefore there is little support for primary hypothesis 2. Not surprisingly working as a nurse at 6 months and 18 months were both significantly associated with working as a nurse at 3 years. Increasing the number of nurses contributing to the logistic regression analysis under the assumption of MAR by simultaneously modelling the independent variables did not change the overall findings.

A finding that was consistent across all models was the increasing strength of relationships the nearer the expressed intentions were to the time-point of interest. Only one moderator variable – ethnicity – had a significant association in the majority of models (4 out of 5). Those who were white British or Black, Asian and Chinese were more likely to intend to work as a nurse in the UK in the future than those from other ethnic groups (other white, white Irish) however nurses who were Black, Asian and Chinese or white Irish were less likely to be working as a nurse at 3 years. Nurses with a spouse or partner at qualification were less likely to be working as a nurse at 18 months than those without, a finding that was not replicated at 3 years.

**Figure 1:**
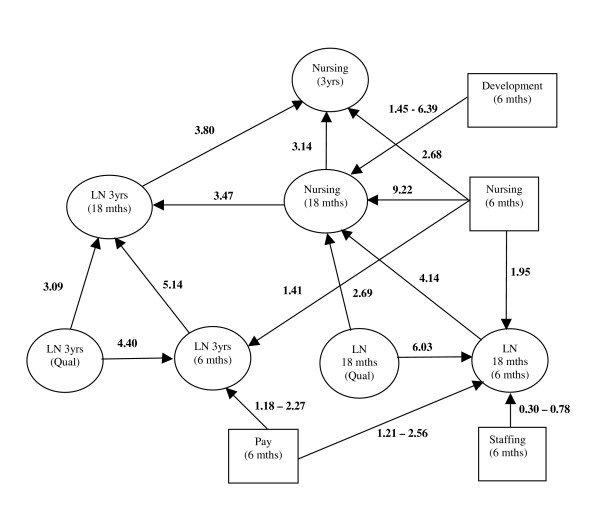
**Path model of job satisfaction, future intentions and nursing at 18 months and 3 years**. Odds Ratios are shown against each path between an exogenous (independent) and an endogenous (dependent) variable. LN denotes the likelihood of nursing (at a time-point in the future). Only those job satisfaction factors that are statistically significant are shown in the diagram and odds ratios are presented for the bottom (1 = very unsatisfied) and top (5 = very satisfied) of the 5 point scale.

We complete the analysis by fitting each of the five models shown in Tables 3 and 4 using first-order job satisfaction factors simultaneously into one structural equation model (Figure 1). Only those first-order job satisfaction factors that were significant are shown. The overall interpretation remains essentially the same.

## Discussion

The findings from this study support the view that intentions are a better predictor of turnover than job satisfaction as shown elsewhere [11] although the latter remains an important 'push factor' in a persons decision to stay or leave an organisation. The strength of relationships in this study increased between intentions and turnover as the gap between each diminished, consistent with previous findings [11].

The data partially support an association between job satisfaction and nursing turnover at 18 months but not at 3 years. The latent variable or second-order factor represented by Development, Relationships and Education had a positive but non-significant association with nursing at 18 months whereas the global effect of these three first-order factors in the logistic regression was significant. Most of this can be attributed to the comparatively strong positive association between turnover and Development and confirms what was previously found by Shields and Ward [27] that training opportunities impacted on turnover more so than workload and pay. The association with Relationships was in the opposite direction. The effect of Relationships, independent of Development, on turnover was weak despite the high correlation between these two variables (r = 0.59). The negative association for Relationship is explained as much by this as anything else. These two factors appear closely connected and development opportunities may be highly dependent on the relationship nurses' have with their line-manager. During the development of the job satisfaction instrument used in this study [13] one of the items, *emotional support from immediate line-manager*, would sometimes (child and mental health branches) load on the development factor rather than the relationships factor.

 Research on job satisfaction and turnover has been contradictory. Some have found that job satisfaction only has an indirect effect on turnover [7] often because the effect of intentions is far stronger or because models have included variables such as organisational commitment which is a more global measure. Lum and colleagues [7] came to the conclusion that nurses who are satisfied with job and pay, are committed to the organisation, and less likely to leave voluntarily.

Job dissatisfaction has been identified as a major predictor of intent to leave [28] and in this study emerges as a predictor of intentions to nurse at 18 months and 3 years but not as strong as intentions expressed at earlier time-points. What is clear from this and other studies is that there is a clear association between certain components of nurses job satisfaction and intention to stay (or leave) the profession or organization [1,5]. The effect of previous intentions, as found before, was stronger the shorter the time gap. Using individual job satisfaction factors provides better predictions than relying on the second-order latent variable representation of the eight job satisfaction factors. Pay satisfaction recorded in first nursing job (six months) was significantly associated with intentions to nurse at 18 months and 3 years. Nurses in this study were more dissatisfied with pay than any other job satisfaction factor [26]. Pay has been found previously to be negatively associated with turnover intent [7,27]. Nurses' perceptions of their own pay in comparison to other public sector occupations can contribute to this dissatisfaction [27] as can a perception of feeling fairly paid or not [29].

Satisfaction with staffing at 6 months was negatively, rather than positively associated with intention to quit at 18 months in this study. Why this particular finding arose in this study is difficult to ascertain. An extensive body of work from the US shows strong associations between dissatisfaction and increasing patient load [30]. Nurses exposed to the full effects of staff shortages for the first time may have led to dissatisfaction at this early stage of career but was not a sufficient reason for leaving nursing during the next 12 months, and paradoxically nurses who were initially dissatisfied with staffing were more likely to nurse in the future. This might be tapping into a social norm whereby nurses feel they should 'battle on' despite the difficulties. At 18 months the association between satisfaction with staffing and intent to quit at 3 years was weak and positive therefore dissatisfaction with staffing, for this cohort, was transitory. Shaver and Lacey [31] identified short staffing as a source of nurses' dissatisfaction and went onto conclude that hospitals paradoxically must employ more nurses to reduce turnover and stipulating minimum nurse-to-patient ratios have been shown to directly benefit turnover [32].

By 18 months development and education have become more important and pay less so. The findings for the SEM and logistic regression models were generally supportive of each other. There may be a connection here with the emergence of development in the intentions model at 18 months (looking ahead to 3 years) and the model for working as a nurse at 18 months.

Ajzen [3] suggests that a behavioural intention can find expression in behaviour only if the behaviour is under volitional control. A persons expectations of obtaining another job has been shown to moderate the relationships between job attitudes and turnover [33]. Findings in Table 2 provide some evidence of the control that nurses have over their decision to remain in nursing. Only a minority of nurses who were unlikely or uncertain about nursing in the future ended up not working as a nurse at a particular time, conversely the vast majority of nurses who were intending to nurse in the future did so. Empirical evidence [2] points to personal considerations overshadowing the influence of social pressure. The latter is likely to vary across occupational groups and pressures exerted on nurses could well be higher than the norm. Moral obligation, which is not included in the theory of planned behaviour, has been found to predict nurses' intentions [34]. Moore [35] believes a sense of professionalism may deter nurses from quitting even when working conditions are difficult and Chang [36] has proposed that individuals who are dually committed to profession and organization are less likely to leave than those committed just to the organisation.

Ethnicity was the only distal variable that was consistently associated with intentions and turnover. Black, Asian and Chinese indicated they were as likely as other groups to nurse in the future however a higher proportion were no longer working as nurses at 3 years. An analysis of NHS nursing staff survey data collected in 1994 [27] also found Black and Asian nurses were more likely to quit. We found no evidence of an association between age, intentions and turnover although associations have been found in other studies [27]. Those with a spouse or partner were less likely to be working as a nurse at 18 months. Nurses' were at the early stage of career and so the impact of, or plans leading to, child bearing would have been minor. There may have been some other reason related specifically to this cohort explaining why this occurred at 18 months and not at 3 years such as movement with partner to another geographical location. Having children living at home was not related to intentions or attrition here but other research has shown children have an affect on decisions to stay or leave nursing [37] and those with children have been shown to have higher levels of job satisfaction [27].

In summary we found strong relationships between intentions and turnover, evidence of a relationship between development and turnover in one model and evidence of relationships between a small number of first-order job satisfaction factors and future intentions with pay being the only factor to emerge on more than one occasion. These findings are in tune with those arising from a meta-analysis of job satisfaction and turnover of nurses conducted by Irvine and Evans [38] although the relationships between job satisfaction and intentions appear to be weaker. They found a small negative relationship between job satisfaction and turnover whereas we found only one significant relationship out of the four tested.

## Limitations

A framework analogous to the theory of planned behaviour was followed. The secondary nature of the analysis limited us to certain variables. We were therefore not able to include variables for subject norms and perceived behavioural control. Future longitudinal work of nurse's early career would therefore benefit from the inclusion of these two salient beliefs.

The data analysed are longitudinal and suffer from attrition. The data were modelled under the assumption of MAR by including moderator variables in the model known to be associated with attrition (e.g. gender, ethnicity). However it is possible that the non-response mechanism was non-ignorable therefore some biases remain. We chose to use an instrument that was specifically developed for nurses in early career. Other instruments may have produced different findings. Some studies [39] have found stronger associations with intent to stay (r = 0.48) than were evident here.

## Conclusion

The aim of this research was to test whether job satisfaction had a direct effect on both intentions and working as a nurse and whether intentions predicted working as a nurse in the future. There was limited evidence for a job satisfaction effect on intentions and working as a nurse. Pay was the only factor, apart from staffing on one occasion, to have a direct effect on intentions. There was partial evidence of a direct effect of job satisfaction on working as a nurse via development that was transitory. Lack of development in early career may well lead to a loss of newly qualified nurses. It has been found that newly qualified nurses reach a low point at around six months [26,40-42] and therefore periods of support should continue beyond this. Evidence from the US [42] suggests that extending the mandatory period of support is a successful way of supporting newly qualified nurses in their transition from student to nurse. This support helps to maintain confidence and job satisfaction leading to longer term benefits that include reduced turnover, improved patient care and reduction in costs. Working as a nurse (actual behaviour) was predicted by intentions, as suggested by the theory of planned [2] behaviour and other empirical research however indirect effects of job satisfaction through intentions are small in size [38]. Additionally, intentions were themselves predicted by their antecedents. We conclude that intentions are a useful marker of future UK nursing and if asked for in a sensitive way could provide a useful source of information both for career development and workforce planning. Finally researchers should be wary of using satisfaction as a proxy for intentions where the goal of the research is to study factors related to turnover.

## Competing interests

This work was undertaken by the National Nursing Research Unit, which receives funding from the Department of Health (DH). The views expressed in this publication are those of the authors and not necessarily those of the DH.

## Authors' contributions

TM participated in study design, was involved in data processing, carried out the analysis, drafted the manuscript and the interpreted the findings. SR made a major contribution to the conception of the study, the design, data collection and interpretation. PG provided intellectual and theoretical input for the paper and interpretation of the findings. All authors were involved in revising the manuscript and have read and approved to final version.
